# Non-pharmaceutical interventions for COVID-19 transiently reduced pneumococcal and *Haemophilus influenzae* carriage in a cross-sectional pediatric cohort in Southampton, UK

**DOI:** 10.1128/spectrum.00224-24

**Published:** 2024-07-11

**Authors:** David W. Cleary, James Campling, Maria Lahuerta, Kyla Hayford, Jo Southern, Bradford D. Gessner, Stephanie W. Lo, Stephen D. Bentley, Saul N. Faust, Stuart C. Clarke

**Affiliations:** 1 Institute of Microbiology and Infection, College of Medical and Dental Sciences, University of Birmingham, Birmingham, United Kingdom; 2 Vaccines Medical Affairs, Pfizer Ltd, Tadworth, United Kingdom; 3 Global Respiratory Vaccines, Scientific and Medical Affairs, Pfizer Inc, Collegeville, Pennsylvania, USA; 4 Evidence Generation, Pfizer Inc, Collegeville, Pennsylvania, USA; 5 Parasites and Microbes, Wellcome Sanger Institute, Hinxton, United Kingdom; 6 Faculty of Medicine and Institute for Life Sciences, University of Southampton, Southampton, United Kingdom; 7 NIHR Southampton Biomedical Research Centre, University Hospital Southampton Foundation NHS Trust, Southampton, United Kingdom; 8 NIHR Southampton Clinical Research Facility, University Hospital Southampton Foundation NHS Trust, Southampton, United Kingdom; 9 Global Health Research Institute, University of Southampton, Southampton, United Kingdom; Griffith University, Gold Coast, Australia

**Keywords:** *Streptococcus pneumoniae*, carriage, COVID-19, SARS-CoV2, *Haemophilus influenzae*

## Abstract

The Southampton pneumococcal carriage study of children under 5 years old continued during the coronavirus disease 2019 (COVID-19) pandemic. Here, we present data from October 2018 to March 2023 describing prevalence of pneumococci and other pathobionts during the winter seasons before, during, and after the introduction of non-pharmaceutical interventions (NPIs) to prevent severe acute respiratory syndrome coronavirus 2 (SARS-CoV-2) transmission. Nasopharyngeal swabs were collected from children attending outpatient clinics at a secondary care hospital and community healthcare sites. Pre-NPIs, in 2019/2020, the carriage prevalence of pneumococci at the hospital site was 32% (*n* = 161 positive/499 participants). During NPIs, this fell to 19% (*n* = 12/64), although based on fewer participants compared to previous years due to COVID-19 restrictions on health-care attendance. In 2021/2022, after NPIs had eased, prevalence rebounded to 33% (*n* = 15/46) [compared to NPIs period, *χ*^2^ (1, *N* = 110) =2.78, *P* = 0.09]. Carriage prevalence at community healthcare sites fell significantly from 27% (*n* = 127/470) in 2019/2020 to 19% during the NPI period (*n* = 44/228) in 2020/2021 [*χ*^2^ (1, *N* = 698) =4.95, *P* = 0.026]. No rebound was observed in 2021/2022 [19% (*n* = 56/288)]. However, in a multivariate logistic regression model, neither site had a significantly lower carriage prevalence during the NPI period compared to the post NPI period. A reduction in serotype diversity was observed in 2020/2021. Carriage of *Haemophilus influenzae* was particularly affected by NPIs with a significant reduction observed. In conclusion, among children under 5 years of age, transient, modest, and statistically non-significant alterations in carriage of both *Streptococcus pneumoniae* and *H. influenzae* were associated with SARS-CoV-2 NPIs.

**IMPORTANCE:**

*Streptococcus pneumoniae* (the pneumococcus) continues to be a major contributor to global morbidity and mortality. Using our long-running pediatric study, we examined changes in pneumococcal carriage prevalence in nearly 3,000 children under the age of 5 years between the winters of 2018/2019 and 2022/2023. This period coincided with the severe acute respiratory syndrome coronavirus 2 pandemic and, in particular, the implementation of national strategies to limit disease transmission in the UK. We observed a transient reduction of both *Streptococcus pneumoniae* and *Haemophilus influenzae* in these populations during this period of non-pharmaceutical interventions. This aligned with the reduction in invasive pneumococcal disease seen in the UK and is therefore a likely contributor to this phenomenon.

## INTRODUCTION

On 2 December 2020, 266 days after the World Health Organization declared the novel coronavirus, severe acute respiratory syndrome coronavirus 2 (SARS-CoV-2), outbreak a pandemic (1), UK regulators granted emergency use authorization for the Pfizer BioNTech (BNT162b2) mRNA vaccine (2). The deployment of other vaccines swiftly followed, for example, ChAdOx1 nCoV-19 (3). Prior to and for some period after implementation of vaccination, non-pharmaceutical interventions (NPIs), i.e., measures independent of drug or specific medical countermeasures, were used to try to reduce transmission, morbidity, and mortality. NPIs included sanitary practices, such as handwashing and cough etiquette, as well as wearing masks, social distancing, travel restrictions and, at the most extreme, enforced stay-at-home periods termed “lockdowns.”

Like most countries, the UK implemented a number of legally binding restrictions to limit social interactions in an attempt to control SARS-CoV-2 transmission (4) that varied in the first 2 years of the pandemic. National lockdowns were first introduced on 16 March 2020, with a strict stay-at-home message. This continued until the beginning of June when, with cases falling, some relaxation allowed groups of up to six people to meet outside, and by the 4 July, most lockdown restrictions had been lifted. In September, it again became illegal to meet in groups of more than six people, and a national “tier” system was introduced in October 2020 to enable restrictions to better reflect regional differences in disease prevalence. Despite these efforts, a second period of national restrictions began on 5 November 2020. An effort to lift restrictions at the beginning of December, again under a tiered system, gradually failed with the introduction of a fourth tier within 2 weeks, a consequence of the emergence of a rapidly spreading SARS-CoV-2 lineage, which later became known as the Alpha variant (5) and ultimately the third national lockdown on 6 January 2021. With vaccines then available, a phased exit from lockdown was implemented from March 2021 with the eventual lifting of all restrictions in July 2021.

Although imperfect, NPIs succeeded in controlling or at least delaying the spread of SARS-CoV-2 (6). As NPIs are non-specific measures, the epidemiology of other respiratory pathogens was also impacted. By using data from the UK’s Health Security Agency (UKHSA), it was shown that the number of notifiable disease cases [including measles, mumps, meningococcal meningitis, scarlet fever, pertussis, food poisoning, and invasive pneumococcal disease (IPD)] decreased by over 80% in England (7) during the early periods of national lockdown. As *Streptococcus pneumoniae*, the pneumococcus, is transmitted via respiratory droplets (8) it is unsurprising that invasive disease and pneumonia associated with this pathogen decreased as a consequence of NPIs, as documented in the UK and across 26 other countries (9, 10). However, data suggest that, although carriage density was impacted in some countries (11), in other areas the proportion of the community that carried this bacterium asymptomatically remained largely unaffected (12–14).

Here, we investigate the impact of UK NPIs on pneumococcal carriage prevalence, as well impact on other common upper respiratory tract pathobionts *Haemophilus influenzae*, *Moraxella catarrhalis*, *Staphylococcus aureus*, and non-pneumococci alpha-hemolytic *Streptococci* in our long-running pediatric carriage study.

## MATERIALS AND METHODS

### Southampton pneumococcal carriage study

The pediatric carriage study has been described extensively. The study started in the winter of 2006/2007 (15, 16) with previous carriage data published up to and including the winter of 2017/2018 (17). Carriage prevalence for other pathobionts relevant to this study has also been previously explored (18). Each year, the target for isolation was *n* = 100 pneumococci. This original number was reached assuming a conservative carriage prevalence of 10% and, therefore, would enable the detection of ~50% relative reduction with 80% power at a 5% significance level.

### Population

Participants were recruited from two sites: University Hospital Southampton (UHS) NHS Foundation Trust (hereafter referred to as Site 1), which serves a population of approximately 1.9 million in Southampton and South Hampshire; a collection of community healthcare facilities within the Solent NHS Trust, covering Portsmouth, Southampton, Hampshire and the Isle of Wight, and included General Practitioners (*n* = 3), health centers/clinics (*n* = 6), and children’s centers (*n* = 7) and serving the same size population but with a focus on regional/community health (referred to as Site 2).

### Swabbing, isolation, and serotyping of pneumococci

Nasopharyngeal swabs were collected from children aged <5 years in the winter (October to March) of 2018/2019 and for each consecutive year until 2022/2023. Parents/guardians were approached for written informed consent either prior to or following their child’s appointment in an outpatient department of Sites 1 or 2. Aside from age, the single exclusion criterion was that only one child per family was swabbed, and that child was swabbed only once per winter season. Nasopharyngeal Rayon Tip Transwabs (Medical Wire, Corsham, UK) in charcoal Amies media were used for swabbing and then plated onto multiple media including CBA (Columbia blood agar with horse blood), CHOC (Columbia blood agar with chocolated horse blood), BACH (Columbia agar with chocolated horse blood and bacitracin), and CNA (Columbia blood agar with colisitin and naladixic acid) (all Oxoid, Basingstoke, UK) within 9 h of swabbing. Confirmation of presumptive *S. pneumoniae* was done on CBA using optochin sensitivity indicated by a 14-mm-diameter inhibition zone around the disc (Thermo Scientific, Loughborough, UK). *H. influenzae* was confirmed as colonies that require both X and V factors on BACH and was done by inoculating the plate with a bacterial suspension in phosphate-buffered saline (PBS) with X, V, and X + V discs (Oxoid, Basingstoke, UK) placed at equal distances. *M. catarrhalis* was confirmed as oxidase-positive, tributyrin-positive, and DNase-positive isolates. Oxidase testing was done by transferring bacterial material onto an oxidase strip (Oxoid, Basingstoke, UK), tributyrin, placing a tributyrin tablet (Sigma-Aldrich, UK) into a 5-mL bacterial suspension in PBS, and DNase by streaking onto a DNase methyl green agar plate (Oxoid, Basingstoke, UK). *S. aureus* was identified as characteristic coagulase-positive colonies using a Pastorex Staph Plus Kit (Bio-Rad, UK). Alpha-hemolytic *Streptococci* were identified as Gram-positive cocci, with incomplete hemolysis on CBA. No further analysis was done on *H. influenzae*, *M. catarrhalis*, *S. aureus*, or non-pneumococcal streptococci and only one colony of *S. pneumoniae* per participant swab was selected for whole-genome sequencing. Pneumococcal isolates from skim milk, tryptone, glucose, and glycerin (STGG) stocks were cultured on CNA plates and incubated overnight at 37°C in 5% CO_2_ prior to DNA extraction. Extraction was carried out using QIAamp DNA mini kit (Qiagen, Hilden, Germany) according to the manufacturer’s instructions. The DNA extracts were sent to the Wellcome Sanger Institute (WSI) for whole-genome sequencing (WGS) using Illumina HiSeq or 10x platforms generating initially 2 × 75 bp and later 2 × 100 bp paired-end reads from libraries prepared using TruSeq chemistry. Pneumococcal serotype was inferred using PneumoCaT version 1.0 (19). As stated above, the goal was to sequence 100 pneumococcal isolates each year. To enable accurate comparisons of pneumococcal serotype prevalence/diversity with previous years, and where pneumococcal isolate numbers exceeded 100, preference was given to sequencing of isolates collected at Site 1. Where numbers were low, these were supplemented with isolates collected from Site 2. For years when less than 100 isolates were available across both sites, e.g., 2020/2021 and 2021/2022, all available pneumococcal isolates were sequenced. Study timelines were such that sequence data were not available for the final year 2022/23 prior to the preparation of this manuscript.

### Viral swabs and SARS-CoV-2 qPCR

An additional swab for the detection of SARS-CoV-2 was added to the study from 2020/2021. Viral RNA was extracted from oropharyngeal (OP) swabs stored at −75°C in viral transport media (VTM; Sigma VIROCULTÒ) using the QIAamp Viral RNA Mini Kit (Qiagen, UK) per the manufacturer’s instructions. An MS2 bacteriophage extraction control (kindly donated by Dr. Nusreen Ahmed, University Hospital Southampton NHS Trust Microbiology Laboratory) was added to each VTM aliquot prior to extraction. Following extraction, each sample underwent four PCR reactions—one for genes N1 and N2 (as a combined reaction) with RNAseP as an internal control (2019-nCoV RUO Kit, IDT, USA), E gene (E Assay_First Line Screening, IDT, USA), and finally MS2 (forward: 5′-TGGCACTACCCCTCTCCGTATTCACG-3′; reverse: 5′-GTACGGGCGACCCCACGATGAC-3′; probe: 5′-FAM-CACATCGATAGATCAAGGTGCCTACAAGC-BHQ1-3′). PCR reactions were run on a Rotor-GENE thermal cycler (Qiagen, UK) with 10 μL of sample, 5 μL of TaqPath mastermix (ThermoFisher, UK), 1.5 μL of each primer for N1/N2 and RNAseP, 2 μL for E, and 3 μL for MS2. After adjusting with dH_2_O, the final reaction volume for each PCR was 20 μL. Positive control materials (2019-CoV Plasmid Controls, IDT, USA) were used at concentrations of 200 copies/μL in place of samples. Reaction conditions were 25°C for 1 min; 50°C for 15 min; 95°C for 2 min followed by 40 cycles of 95°C for 3 s, 60°C for 30 s. Quantification was done using a standard curve generated by extraction of the World Health Organization SARS-CoV-2 RNA standard at concentrations of 10^7^ to 10^0^ in 140 μL of VTM.

### Statistical Analysis

All statistical analysis was done in R version 4.2.2 (2022–10-31) using RStudio version 2022.12.0+353 with graphics built using the grammar of graphics package, ggplot2 (20, 21). Children were grouped by age into 0–6, 6–11, 12–23, 24–35, 36–47, and 48–60 months. The first three categories (0–6, 6–11, and 12–23 months) are considered the most meaningful in relation to pneumococcal carriage and the influence of the childhood immunization schedule in place prior to COVID-19. Moreover, these categories enable meaningful comparisons to previously published data from this cohort. The pre-NPI period was defined as any sample collected on or before 16 March 2020, with 17 March 2020 to 1 July 2021 denoting the NPI period and post-NPI period being on or after 1 August 2021. Chi-squared tests were used to examine pneumococcal carriage prevalence between sampling years. Simpsons index of diversity was computed for pneumococcal serotypes using the diversity() function from the R package vegan (22). To account for differences in sampling between pre-NPI and during-/post-NPI years, where in the latter, recruitment was generally lower, this was done after rarefying each year to an even sampling depth (*n* = 50). Odds ratios based on univariate and multivariable logistic regression analysis were done using the R package finalfit() and glmulti(). Both were reported given that it was unclear whether NPIs may impact the general features (age, gender, etc.) of the population recruited to the study. Thus, while multivariate analysis would ordinarily be sufficient to account for complex interactions of carriage predication, inter-period comparisons were not considered immune to these sampling differences. Data were only used in models where questionnaire data were complete for age, gender, and reported vaccine use to minimize reporting inaccuracies. For the model, the dependent variable was carriage, the explanatory variables a character list of age group, whether the household had experienced a case of SARS-CoV-2 infection in the previous 30 days, and NPI period.

## RESULTS

**Fig 1:**
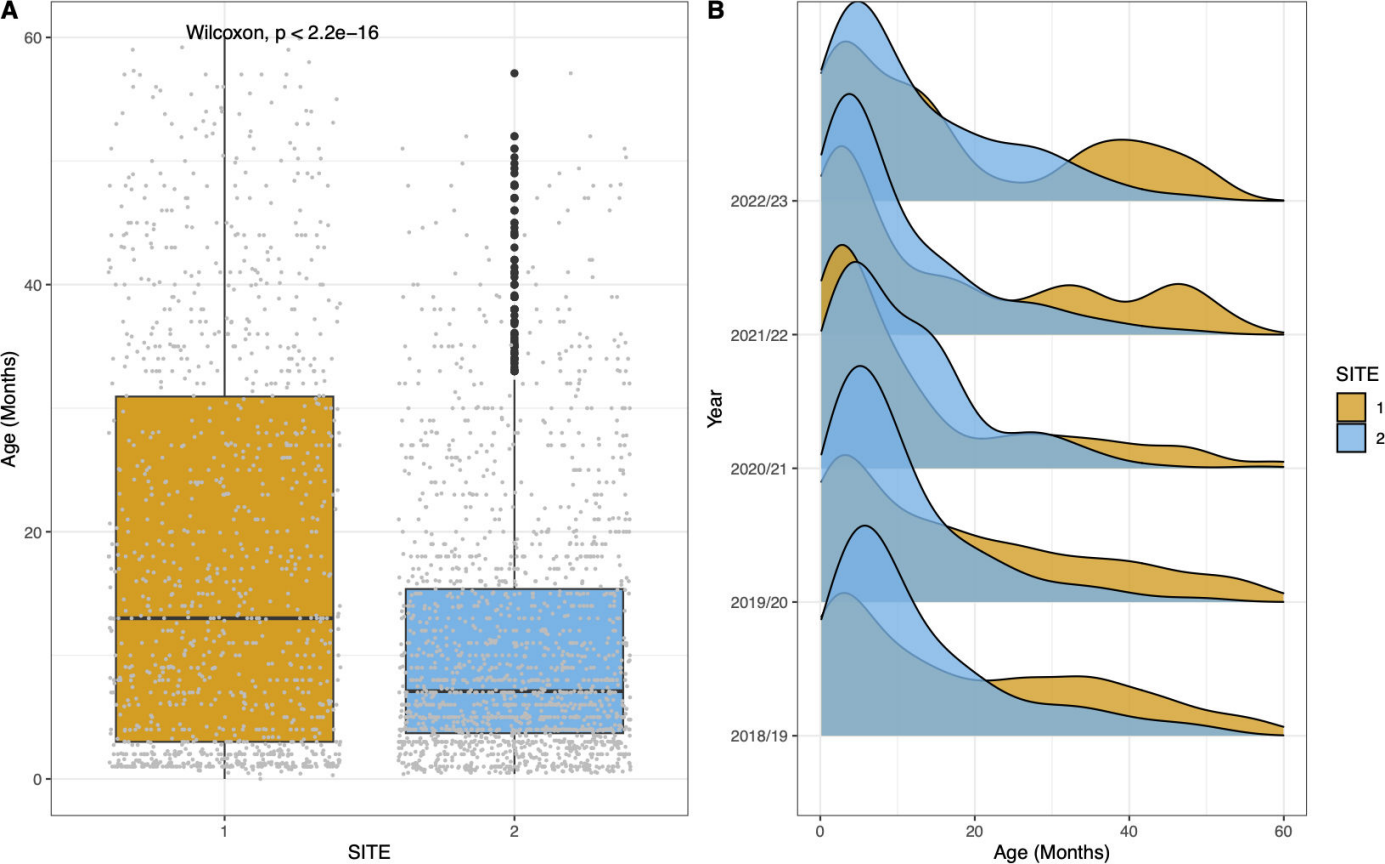
Box and whisker plot showing the comparison of child age between sites (**A**) and density plot showing distribution across age for each site by recruitment year (**B**). Sites are colored: orange (Site 1, hospital) and blue (Site 2, community clinics). Boxes show the interquartile range with whiskers denoting minimum and maximum values. Outliers are shown as black points. Individual data points are shown in gray. Children recruited at Site 1 between 2017/2018 and 2022/2023 were significantly (*P* < 0.001) older than those at Site 2.

A total of 2,966 children under the age of 5 years were recruited to the study between the winters of 2018/2019 and 2022/2023. Recruitment numbers per year, with age, gender, and point prevalence of pneumococcal carriage are shown in Table 1. At Site 1 recruitment numbers ranged from a low of *n* = 31 in 2022/2023 to *n* = 542 in 2018/2019. At Site 2, the lowest recruitment was in 2020/2021 with *n* = 228 and the highest with *n* = 470 in 2019/2020. Most children (*n* = 1784, 60.1%) were recruited from Site 2. There was no significant difference between sites in the ratio of males to females (*P* = 0.35, row-wise *z*-test of proportions) with males accounting for 43% (*n* = 513/1,182) and 46% (*n* = 820/1,784) of the populations, respectively, at Sites 1 and 2. As shown in Fig. 1, there was a significant (*P* < 0.001) difference in age between sites. The mean age of children recruited in Site 1 was 18.1 months (± 16.37; range: 0–59.9) compared to 11.1 months (±10.5; range: 0.4–57.1) at Site 2 (Fig. 1A). There were also clear differences in age at recruitment between years at each site, as shown in Fig. 1B.

**TABLE 1:** Number of children recruited each year (2017/2018 to 2022/2023) at each site (Site 1—hospital, Site 2—community clinics), showing age (minimum, maximum, mean, and standard deviation), gender, and carriage of *S. pneumoniae^a^*

	Pre-NPI				During NPI		Post-NPI			
	(2018/19)		(2019/20)		(2020/21)		(2021/22)		(2022/23)	
	Hospital Site 1	CHC Site 2	Hospital Site 1	CHC Site 2	Hospital Site 1	CHC Site 2	Hospital Site 1	CHC Site 2	Hospital Site 1	CHC Site 2
N	542	458	499	470	64	228	46	288	31	340
Age										
Min	1	0.5	0.3	0.5	0.47	0.4	0	0.5	0.75	0.5
Max	59	52	59.93	50.3	59.2	57.1	53	48	49	48
Mean (SD)	18.93 ± 16.49	12.71 ± 11.56	18.18 ± 16.28	9.93 ± 9.40	12.32 ± 14.80	10.78 ± 9.49	16.51 ± 16.91	9.43 ± 9.92	17.35 ± 16.45	12.30 ± 10.94
Gender										
Male (%)	209 (39)	203 (44)	231 (46)	215 (46)	34 (53)	119 (52)	25 (54)	138 (48)	14 (45)	145 (43)
Female (%)	202 (37)	220 (48)	246 (49)	220 (47)	30 (47)	109 (48)	21 (46)	146 (51)	16 (52)	194 (57)
Unknown (%)	131 (24)	35 (8)	22 (4)	35 (7)	0 (0)	0 (0)	0 (0)	4 (1)	1 (3)	1 (0)
*S. pneumoniae*										
Positive (%)	192 (35)	158 (34)	161 (32)	127 (27)	12 (19)	44 (19)	15 (33)	56 (19)	13 (42)	86 (25)
Negative (%)	350 (65)	300 (66)	338 (68)	343 (73)	52 (81)	184 (81)	31 (67)	232 (81)	18 (58)	254 (75)

^*a*^ CHC, community healthcare sites; NPI, non-pharmaceutical interventions.

To determine the impact of COVID-19 infection on pneumococcal carriage, we screened participants for SARS-CoV-2 using qPCR for several viral targets. Infection in this instance was either early (pre-symptomatic) or asymptomatic. In 2020/2021, the first year of viral swabbing, 198/292 participants (67.8%) consented to the additional swab, from whom no positives were identified. In 2021/2022, participation in viral swabbing rose to 89.2% (*n* = 298/334). From these, nine had weak PCR positives that did not meet the threshold of a *Ct* <30 for a valid positive. One participant, a 5-month-old infant, was positive for N1, N2, and E gene PCRs with *Ct* values <30. No SARS-CoV-2 had been reported in the household, although the child was reported to have had an ear and throat infection in the previous 30 days. The infant was not a pneumococcal carrier, with only *M. catarrhalis* isolated. One further child aged 7 months was also positive with a *Ct* value of <30 for both N and E targets with no reported respiratory symptoms, either personally or in the household, and was a pneumococcal carrier. In 2022/2023, participation in viral swabbing decreased to 71.0% (*n* = 264/372). Weak PCR positives that did not meet the threshold of a *Ct* <30 for a valid positive accounted for 11.7% of swabbed children (31/264). A further nine (3.4%) had qPCR positives with <30 *Ct* for N but not E gene targets even after multiple re-extractions and re-tests and therefore were not classed as positives. Only two children had Ct values <30 for both N and E. They were both 7 months old. One child had no reported respiratory infection in the past 30 days and no history of SARS-CoV-2 infection, either personally or in a household contact. The other child had tested positive 5 months prior to swabbing, had a household contact who also tested positive at the same time, and had cold-like symptoms in the 30 days to prior to recruitment. Stored STGG samples from Site 1 were also tested from 2019/2020, noting that recruitment would have been during the very early phase of the pandemic in the UK. A number (*n* = 95) failed either with extraction or PCR control issues; however, given the limited stored sample volumes, it was not possible to re-test. From the remaining samples (*n* = 404) no positives were identified.

**Fig 2:**
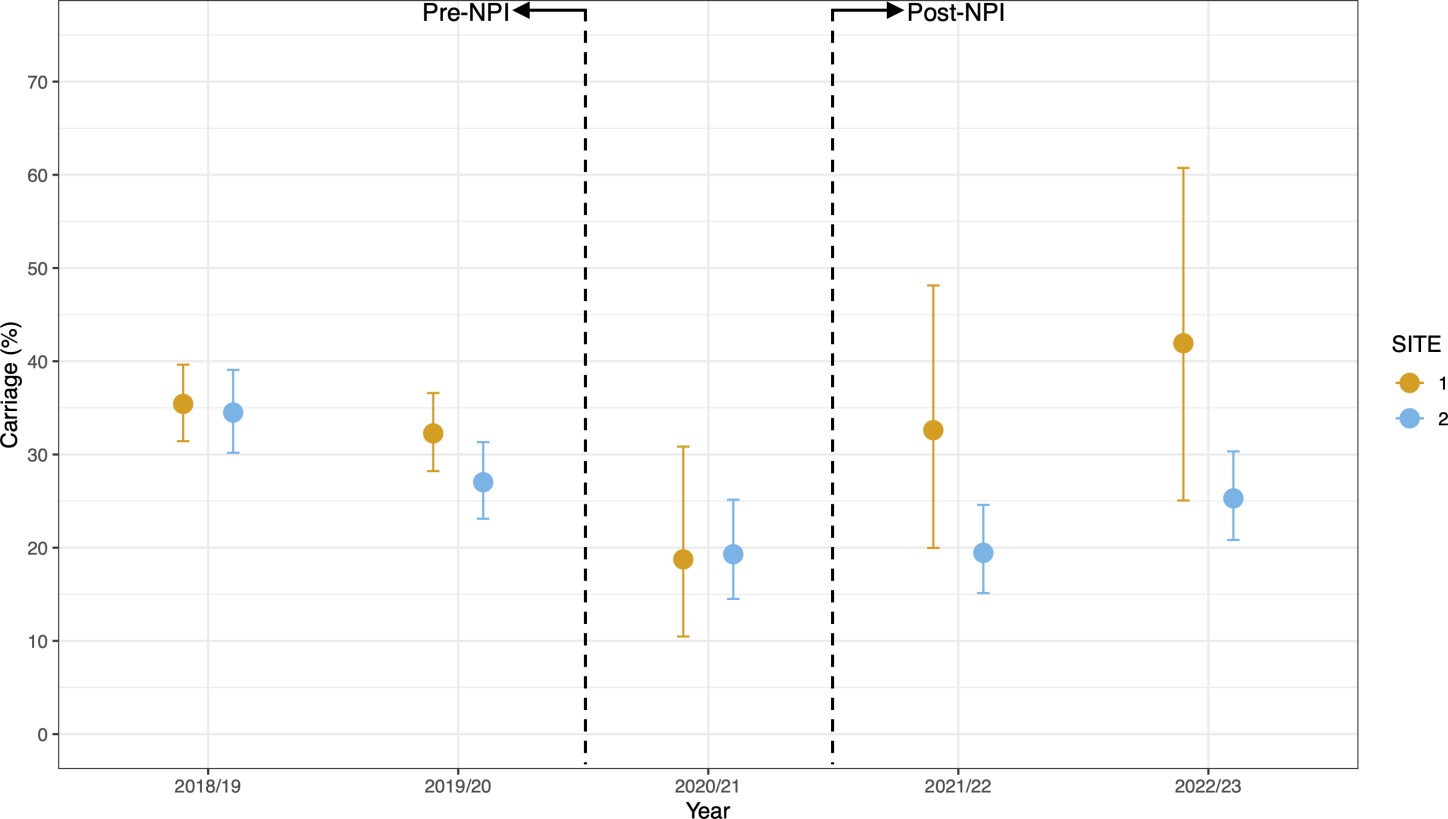
Carriage prevalence of *S. pneumoniae* shown for each year of the study and split by Site 2 (hospital, orange) and Site 2 (community clinics, blue). The dashed lines highlight the pre-NPI and post-NPI periods. Only one period of recruitment was done within the period of NPI lockdowns in the UK, 2020/2021. Carriage prevalence at Site 2 decreased significantly from 27% (*n* = 127/470) in 2019/2020 to 19% *[n* = 44/228) in 2020/2021 (χ^2^ (1, *N* = 698) =4.95, *P* = 0.026)]. No immediate rebound was observed in 2021/2022, but by 2022/2023, carriage was closer to pre-NPIs. Error bars show 95% CI.

Pneumococcal carriage ranged from 19% (*n* = 44/228) to 42% (*n* = 13/31) at Site 1 and 19% (*n* = 56/288) to 34% (*n* = 158/458) at Site 2. Carriage prevalence across all years is shown in Fig. 2. A decrease in pneumococcal carriage was observed during the period of NPIs impacting year 2020/2021. In 2019/2020, pre-pandemic, carriage prevalence at site 1 was 32% (*n* = 161/499), which decreased in 2020/2021 to 19% (*n* = 12/64), although based on notably fewer children being recruited. In 2021/2022, after NPIs had eased, prevalence rebounded to 33% (*n* = 15/46). Although this increase was not significant when comparing proportion to the previous year [χ^2^ (1, *N* = 110) =2.78, *P* = 0.09)], it was nevertheless more reflective of pre-lockdown periods and was followed by a 42% (*n* = 13/31) carriage prevalence in 2022/2023. In comparison, carriage prevalence at Site 2 fell significantly from 27% (*n* = 127/470) in 2019/2020 to 19% (*n* = 44/228) in 2020/2021 [χ^2^ (1, *N* = 698) =4.95, *P* = 0.026)]. No immediate rebound was observed in 2021/2022 [19% (*n* = 56/288)], but by 2022/2023, carriage was closer to pre-NPIs levels at 25% (*n* = 86/340). Using univariate and multivariate binary logistic regression analysis (Table 2), we saw, as expected based on our previous data published on this cohort (17), that pneumococcal carriage was significantly associated with age at both sites, with older age associated with higher odds. In the univariate model, the odds ratio of pneumococcal carriage during NPIs was lower in both Site 1 (OR 0.51: 0.26–0.95 *P* = 0.044) and Site 2 (OR 0.54: 0.37–0.77 *P* = 0.001) and remained lower in Site 2 post-NPIs (OR 0.68: 0.53–0.86 *P* = 0.002). However, these associations were not significant in the multivariate model. Interestingly, children recruited at Site 2 from households that had a SARS-CoV-2 infection in the previous 30 days were more likely to carry pneumococci (OR 1.51: 1.08–2.11 *P* = 0.015), but again, this was not significant in the multivariate model.

**TABLE 2:** Odds ratio for pneumococcal carriage by recruitment site (Site 1—hospital, Site 2—community clinics) using univariable and multivariable binary logistic regression^*a*^

Dependent variable: *S. pneumoniae* carriage		Negative N (%)	Positive N (%)	OR (univariable)	OR (multivariable)
Site 1					
Age group	<6	304 (81.3)	70 (18.7)	–	
	7–11	70 (64.2)	39 (35.8)	**2.42 (1.51–3.86, *P* < 0.001**)	1.89 (0.46–7.78, *P* = 0.378)
	12–23	102 (55.1)	83 (44.9)	**3.53 (2.40–5.23, *P* < 0.001)**	2.30 (0.69–7.68, *P* = 0.178)
	24–35	83 (56.1)	65 (43.9)	**3.40 (2.25–5.16, *P* < 0.001)**	3.57 (0.91–14.04, *P* = 0.069)
	36–47	69 (56.1)	54 (43.9)	**3.40 (2.19–5.29, *P* < 0.001)**	**6.97 (1.68–28.92, *P* = 0.007)**
	48–60	42 (72.4)	16 (27.6)	1.65 (0.86–3.06, *P* = 0.118)	1.73 (0.27–11.20, *P* = 0.564)
SARS CoV2 in household	No	41 (68.3)	19 (31.7)	–	–
	Yes	26 (60.5)	17 (39.5.6)	1.41 (0.62–3.21, *P* = 0.410)	1.03 (0.40–2.67, *P* = 0.957)
NPI period	Pre-lockdowns	578 (66.7)	288 (33.3)	–	–
	During-lockdowns	47 (79.7)	12 (20.3)	**0.51 (0.26–0.95, *P* = 0.044)**	–
	Post-lockdowns	44 (62.0)	27 (38.0)	1.23 (0.74–2.02, *P* = 0.414)	1.42 (0.50–4.02, *P* = 0.513)
Site 2					
Age group	<6	666 (84.4)	123 (15.6)	–	–
	7–11	223 (69.3)	99 (30.7)	**2.40 (1.77–3.26, *P* < 0.001)**	**2.96 (1.76–4.98, *P* < 0.001)**
	12–23	210 (63.3)	122 (36.7)	**3.15 (2.34–4.23, *P* < 0.001)**	**5.07 (3.24–7.95, *P* < 0.001)**
	24–35	99 (62.7)	59 (37.3)	**3.23 (2.21–4.69, *P* < 0.001)**	**4.58 (2.69–7.81, *P* < 0.001)**
	36–47	24 (51.1)	23 (48.9)	**5.19 (2.83–9.51, *P* < 0.001)**	3.61 (1.31–9.92, *P* = 0.013)
	48–60	5 (35.7)	9 (64.3)	**9.75 (3.31–32.18, *P* < 0.001)**	*
SARS CoV2 in household	No	359 (81.4)	82 (18.6)	–	–
	Yes	284 (74.3)	98 (25.7)	**1.51 (1.08–2.11, *P* = 0.015)**	1.25 (0.85–1.85, *P* = 0.262)
NPI period	Pre-lockdowns	579 (69.6)	253 (30.4)	–	–
	During-lockdowns	182 (80.9)	43 (19.1)	**0.54 (0.37–0.77, *P* = 0.001)**	–
	Post-lockdowns	467 (77.1)	139 (22.9)	**0.68 (0.53–0.86, *P* = 0.002)**	1.23 (0.78–1.95, *P* = 0.378)

^*a*^ The dependent variable was carriage, with explanatory variables being age group, recent household SARS-CoV2 infection, and NPI period. Significant results are shown in bold. * denotes an infinite OR value given few data points for that group.

**Fig 3:**
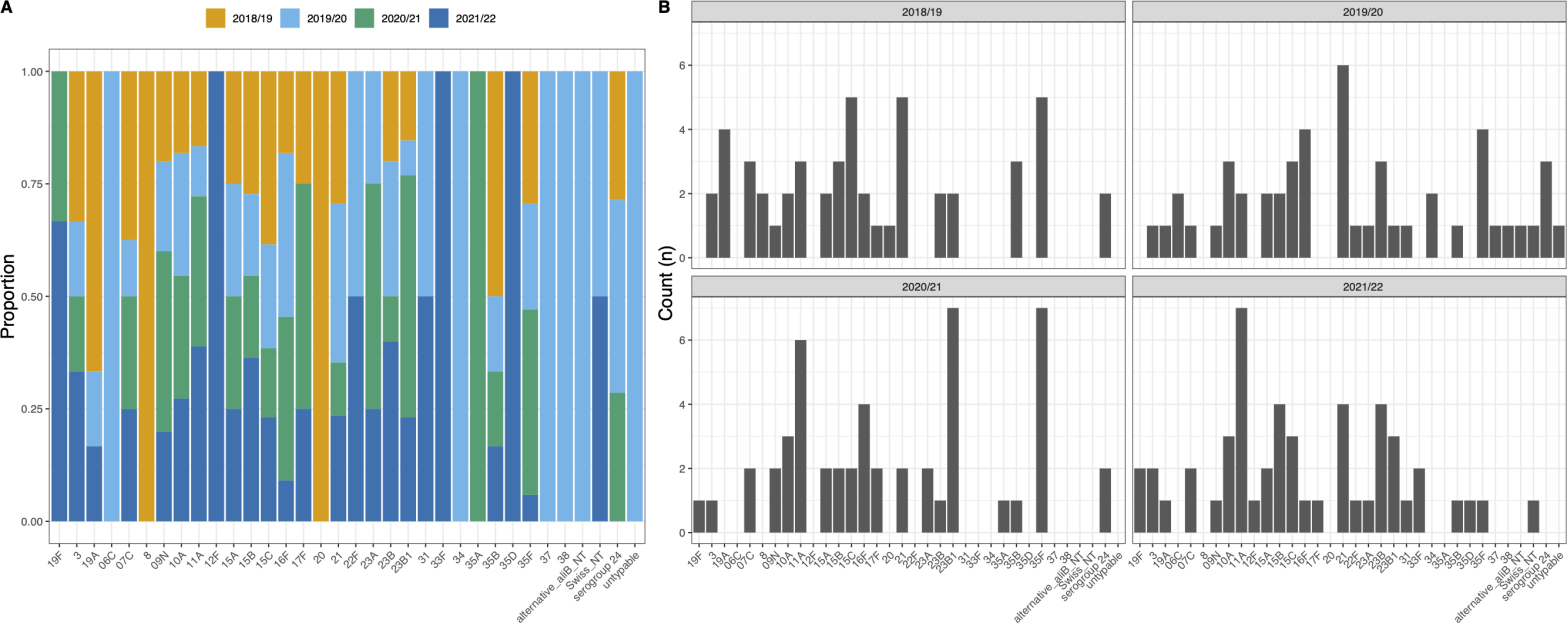
Pneumococcal serotypes isolated (*n* = 35) shown as a per-year proportion (**A**) with individual counts for each serotype split by year (**B**). Data presented are based on a subsample of isolates from each year (*n* = 50) to allow for meaningful comparisons between pre-NPI years and during-NPI periods when both recruitment and pneumococcal carriage was lower. Only three vaccine-type serotypes were isolated (19F, 3, and 19A) and were isolated each year apart from 2020/2021 when 19A was not observed. Fewer serotypes were seen during the NPI period (*n* = 19) compared to both pre- and post-NPI periods.

Serotype prevalence as a per-year proportion is shown in Fig. 3A with counts shown in Fig. 3B. Over the 4-year period for which we had data, a total of 35 unique serotypes were identified (including three non-typeable/untypable groups). Pre-NPIs, 28 and 30 unique serotypes in each year were identified for 2018/2019 and 2019/2020, respectively. In 2020/2021, this dropped to *n* = 19, and in 2021/2022, it was *n* = 24. This resulted in a reduced diversity with Simpson’s indexes of 0.918, 0.913, 0.898, and 0.902. Importantly, these were calculated using an even sampling depth of *n* = 50 isolates for each year to account for biased sampling between years. The most commonly identified serotype was 21A, which accounted for 9% (*n* = 43/479) and ranked as the most common serotype in 2018 to 2020, and then sixth and fourth in 2020/2021 and 2021/2022, respectively. Most serotypes were found in at least 2 years with only eight appearing in only one: serotypes 8 and 20 in 2018/2019, serotypes 6C, 34, and 37 in 2019/2020, serotype 12F in 2020/2021, and serotypes 35D and 35A in 2021/2022. It is worth noting that post-NPIs, there was consistency in the prevalence of common serotypes compared to pre-NPIs. This is best reflected with 10A and 11A, which accounted for 7.1% and 9.4% of all isolates, respectively, and were in the five most commonly identified serotypes in each year, apart from in 2019/2020 where 11A dropped to seventh (5.1%; *n* = 8/156). Low-level PCV13 vaccine-type carriage remained post-NPIs with serotypes 19A, 3, and 19F all seen in each year. The exception was 2019/2020 where 19A was not observed.

**Fig 4:**
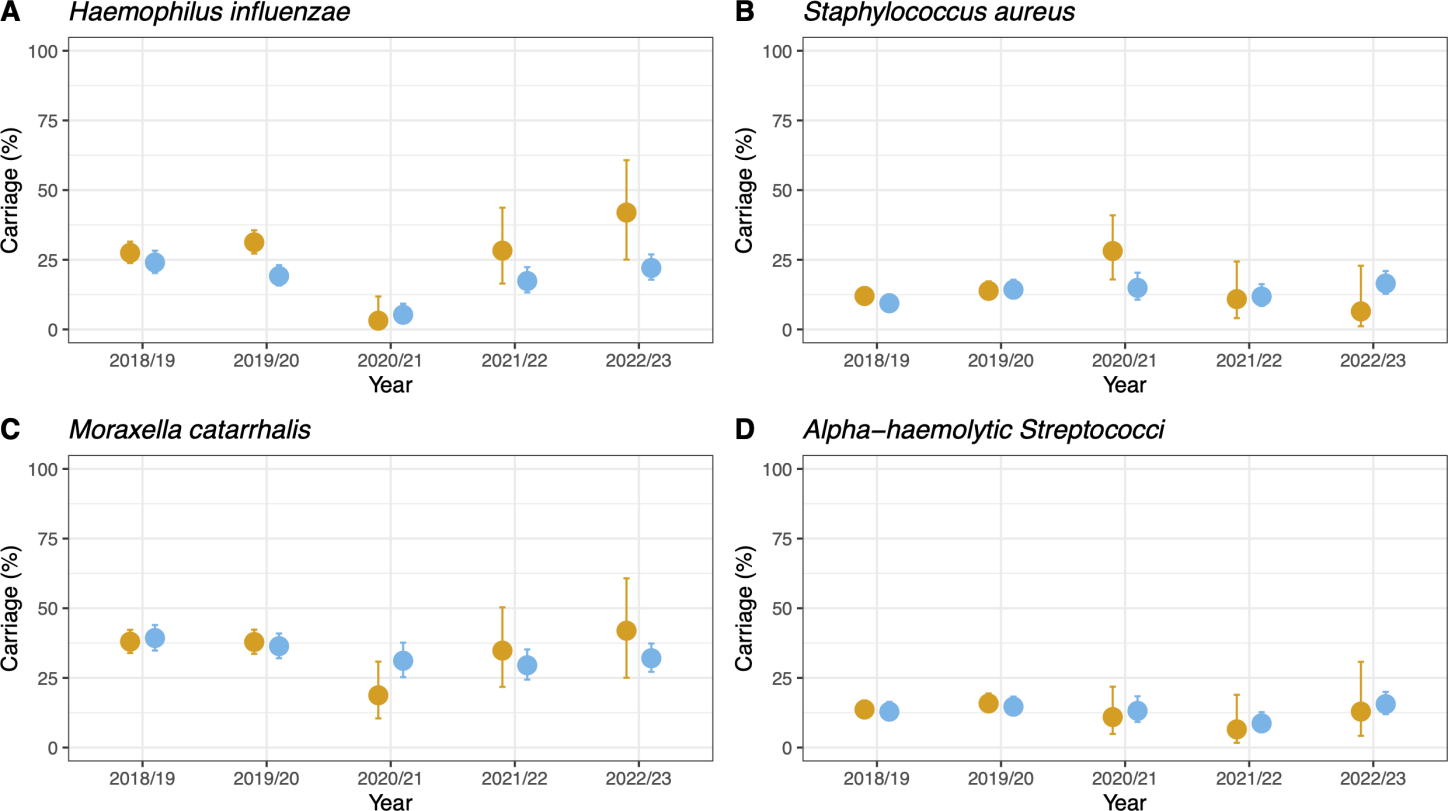
Carriage prevalence of *H. influenzae* (**A**), *S. aureus* (**B**), *M. catarrhalis* (**C**), and non-pneumococci alpha-hemolytic *Streptococci* (**D**). Carriage for each pathobiont in each year is shown split by Site 1 (hospital, orange) and Site 2 (community clinics, blue). Error bars show 95% CI. The only significant decrease in carriage was observed for *H. influenzae* during the NPI period (2020/021) where carriage decreased to 3.1% (*n* = 2/64) and 5.3% (*n* = 12/228) for Sites 1 and 2, respectively.

We next examined carriage of other common bacteria of the upper respiratory tract (Fig. 4A through D). There appeared to be little impact on carriage of *S. aureus* (Fig. 4B), *M. catarrhalis* (Fig. 4C), or non-pneumococcal alpha-hemolytic *Streptococci* (Fig. 4D). However, there was a clear reduction in *H. influenzae* carriage during the NPI period, which was observed in children recruited at both sites (Fig. 4A). In the year preceding NPIs, carriage was 31.3% (*n* = 156/499) and 19.1% (*n* = 90/470) for Sites 1 and 2, respectively. This dropped 3.1% (*n* = 2/64) and 5.3% (*n* = 12/228) during NPIs (2020/2021) before rebounding in 2021/2022 to 28.3% (*n* = 13/46) at Site 1 and 17.4% (*n* = 20/288) at Site 2. Using the same model set-up as used for pneumococcal carriage, we observed increasing odds of *H. influenzae* carriage as age increased (Table 3). A decrease in odds for carriage in the univariate model was seen pre-NPIs to during NPIs (OR 0.08: 0.01–0.26, *P* < 0.001 and 0.20: 0.11–0.36, *P* < 0.001) for Sites 1 and 2, respectively. As for pneumococci, there was an increased odds of carrying *H. influenzae* in children from households that had reported a SARS-CoV-2 episode in the previous 30 days, although this was not significant in the multivariate model (Table 3).

**TABLE 3:** Odds ratio for carriage of *H. influenzae* by recruitment site (Site 1—hospital, Site 2—community clinics) using univariable and multivariable binary logistic regression^*b*^

Dependent variable: *H. influenzae* carriage		Negative N (%) 2058 (77.3)	Positive N (%) 604 (22.7)	OR (univariable)	OR (multivariable)
Site 1—hospital (*N* = 999^*a*^)					
Age group	<6	322 (86.1)	52 (13.9)	-	-
	7–11	74 (67.9)	35 (32.1)	**2.93 (1.77–4.81, *P* < 0.001)**	1.45 (0.22–9.69, *P* = 0.701)
	12–23	120 (64.9)	65 (35.1)	**3.35 (2.21–5.13, *P* < 0.001)**	1.17 (0.23–5.89, *P* = 0.850)
	24–35	87 (58.8)	61 (41.2)	**4.34 (2.80–6.76, *P* < 0.001)**	**6.97 (1.38–35.29, *P* = 0.019)**
	36–47	63 (51.2)	60 (48.8)	**5.90 (3.74–9.38, *P* < 0.001)**	**8.95 (1.81–44.32, *P* = 0.007)**
	48–60	41 (70.7)	17 (29.3)	**2.57 (1.33–4.80, *P* = 0.004)**	5.82 (0.79–42.88, *P* = 0.084)
SARS CoV2 in household	No	52 (83.9)	10 (16.1)	-	-
	Yes	28 (63.6)	16 (36.4)	**2.97 (1.21–7.62, *P* = 0.020)**	1.53 (0.51–4.56, *P* = 0.446)
NPI period	Pre-NPI	602 (69.5)	264 (30.5)	-	-
	During-NPI	57 (96.6)	2 (3.4)	**0.08 (0.01–0.26, *P* < 0.001)**	-
	Post-NPI	46 (64.8)	25 (35.2)	1.24 (0.74–2.04, *P* = 0.408)	**14.96 (1.76–127.30, *P* = 0.013)**
Site 2—CHC (*N* = 1,663^*a*^)					
Age group	<6	711 (90.1)	78 (9.9)	-	-
	7–11	266 (82.6)	56 (17.4)	**1.92 (1.32–2.78, *P* = 0.001)**	1.29 (0.69–2.43, *P* = 0.425)
	12–23	232 (69.9)	100 (30.1)	**3.93 (2.83–5.48, *P* < 0.001)**	**3.53 (2.16–5.77, *P* < 0.001)**
	24–35	109 (69.0)	49 (31.0)	**4.10 (2.71–6.17, *P* < 0.001)**	**3.76 (2.13–6.63, *P* < 0.001)**
	36–47	26 (55.3)	21 (44.7)	**7.36 (3.93–13.69, *P* < 0.001)**	**3.62 (1.29–10.12, *P* = 0.014)**
	48–60	5 (35.7)	9 (64.3)	**16.41 (5.53–54.54, *P* < 0.001)**	3.14 (0.30–32.74, *P* = 0.339)
SARS CoV2 in household	No	384 (87.1)	57 (12.9)	-	-
	Yes	306 (80.1)	76 (19.9)	**1.67 (1.15–2.44, *P* = 0.007)**	1.02 (0.67–1.54, *P* = 0.937)
NPI period	Pre-NPI	652 (78.4)	180 (21.6)	-	-
	During-NPI	213 (94.7)	12 (5.3)	**0.20 (0.11–0.36, *P* < 0.001)**	-
	Post-NPI	485 (80.0)	121 (20.0)	0.90 (0.70–1.17, *P* = 0.443)	**4.78 (2.47–9.24, *P* < 0.001)**

^*a*^ Sample number used in models. CHC, community healthcare sites; NPI, non-pharmaceutical interventions.

^*b*^ The dependent variable was carriage, with explanatory variables being age group, recent household SARS-CoV2 infection, and NPI period. Significant results are shown in bold.

Finally, given the recognized interactions between the two species in the upper airways (23), we explored if co-carriage of pneumococci and *H. influenzae* had also been impacted. Here, again, a notable decrease in co-carriage was observed during NPIs (Fig. S1) where in the pre-NPI period, co-carriage at both Sites 1 and 2 was 11.5%, decreasing to 3% and 2%, respectively, in the NPI period. After NPI co-carriage rebounded to an average of 26.5% at Site 1 and 8.5% at Site 2, although, again, we highlight the lower numbers of participants recruited at Site 1 during this period. We noted reductions in carriage also in those only carrying one of these two species, again more pronounced in *H. influenzae* carrying children, but irrespective of pneumococcal co-carriage (Fig. S2), which suggests there was an interaction which drove NPI-related carriage reductions in both. Nevertheless, in general, this translated to an increased OR of co-carriage in the post-NPI period, compared to during NPIs, of 4.90 (2.24–12.34, *P* < 0.001) and 4.79 (2.03–13.26, *P* = 0.001) at the two sites respectively.

## DISCUSSION

During the COVID-19 pandemic, NPIs proved effective at reducing or delaying SARS-CoV-2 transmission and disease (24). These interventions also reduced disease from other respiratory pathogens (9). At least for IPD, earlier studies reported that the dynamic of this reduction was more linked to the control of other seasonal viruses rather than as a consequence of any change in pneumococcal carriage *per se* (14). Using the long-running, cross-sectional pediatric pneumococcal carriage study in Southampton, UK, we sought to determine whether any changes in carriage took place. We also looked at serotype prevalence and diversity as well as the carriage of other pathobionts. Although we observed no long-lasting changes in pneumococcal carriage, in keeping with data from Belgium and France (12, 13), we found a transient reduction during the NPI period, similar to that observed in brief, post-NPI periods in Israel (14) and Vietnam (11). However, we could not attribute this to any one serotype, as in Vietnam, nor to non-encapsulated pneumococci.

Our finding of a reduction and swift rebound in pediatric *H. influenzae* carriage during the COVID-19 pandemic has not been described elsewhere and raises some interesting questions. For example, it is unclear why this pathobiont was more impacted than others, such as *M. catarrhalis*. For comparison, a study of younger children, aged 3–36 months, in the USA noted a decrease in both *H. influenzae* and *M. catarrhalis* during the pandemic, but not pneumococci (25) as did a recent study of South African children <60 months old (26). We have previously shown in our cohort that carriage of *H. influenzae* increases with age (18). As such, perhaps it is possible that school attendance disruption was more consequential for older children in the context of *Haemophilus* transmission, as in the UK children aged 4 years old usually attend school in the year in which they turn 5 years and therefore would be eligible for study participation. All age groups experienced a drop in carriage, but additional stratified analysis was made difficult by the limited recruitment (*n* = 3) of children aged 49–60 months old in the NPI period. Previous studies have highlighted the importance of transmission in child–primary caregiver dyads (27), and therefore, the child–adult transmission link may be more important here.

The rapid rebound in carriage has been mirrored to some extent in disease. For example, annual incidences of *H. influenzae* disease, as recorded by Public Health Scotland, decreased to 0.93 cases per 100,000 in 2020 and 2021 but were back to 1.35/100,000 in 2022, more in keeping with 2018 (1.51) and 2019 (1.52) rates (28). Similar observations were made in England with cases rebounding in 2022 by 57% in comparison to 2021, but still with a low incidence (1.1/100,000) (29). Similarly, in France, children under 5 years old showed the greatest increase in invasive *Haemophilus* disease following relaxation of restrictions, driven primarily by serotype b and, to a lesser extent, serotype a, although it was unclear if this was due to a rebound in circulation, an immunity gap caused by lower vaccine uptake or both (30). Data from China suggest a persistent reduction in childhood respiratory disease caused by *H. influenzae*, but not bacteremia or meningitis, which impacted older children only (31). The authors of this study postulated this was due to an interruption in community transmission caused by NPIs. It is possible that the differences in NPI impact on respiratory pathobionts is a consequence of carriage duration variability, with those organisms experiencing regular turnover potentially being impacted more. Certainly, *H. influenzae* in children has been shown to be subject to frequent strain loss/acquisition (32); however, carriage duration and turnover for pneumococci has been shown to be similar (33). There is a paucity of data related to duration of *Moraxella* sp. carriage in healthy children, but again, the dynamic has been shown to be characterized as one of regular strain turnover (34). Unfortunately, it is impossible to determine exactly what drove the reduction using the data from this study but instead highlights an area for future exploration.

This study had limitations. We did not explore changes in carriage density, which, as discussed, were linked to NPIs in other countries (11). The restrictions on hospital visits during the pandemic resulted in the lower recruitment at Site 1 compared to those of previous years. Ultimately, this meant that meaningful comparisons at this site alone were not possible, which otherwise would have been a strength of the study given how long it had been running. Although we were able to recruit in other community healthcare settings, there may have been different demographics of sampling. We also do not report serotype data for 2022/2023; however, given the serotypes observed in 2021/2022, we do not expect to have missed any NPI-related impacts in prevalence and/or diversity. In all such studies, estimating changes in community-level carriage from a proxy population is an imperfect approach and is subject to potential geographic and demographic biases. Finally, through the examination of only one pneumococcal isolate per participant, it was not possible to examine whether co-carriage of multiple serotypes was impacted. Even accepting these limitations, the main strength of this study is that it is one of the longest running, cross sectional study of pneumococcal carriage of its kind and is one of the few data sets to examine prospectively the impacts of NPIs against existing baseline data.

In conclusion, there was a transient and modest reduction in both serotype non-specific pneumococcal and *H. influenzae* carriage during the period of NPIs in our pediatric cohort. This was accompanied by a reduced diversity of circulating pneumococcal serotypes. Long-lasting impacts of NPIs on pathobiont carriage among children were not observed.

## Data Availability

All sequencing data (fastqs) have been deposited in the European Nucleotide Archive under study accession PRJEB2417 (whole-genome sequencing of carried *Streptococcus pneumoniae* during the implementation of pneumococcal conjugate vaccines in the UK).

## SUPPLEMENTAL MATERIAL

Figure S1 spectrum.00224-24-s0001.tiff Prevalence of *Streptococcus pneumoniae* and *Haemophilus influenzae* co-carriage.

Figure S2 spectrum.00224-24-s0002.tiff Carriage prevalence of *Streptococcus pneumoniae* and *Haemophilus influenzae*.

ASM does not own the copyrights to Supplemental Material that may be linked to, or accessed through, an article. The authors have granted ASM a non-exclusive, world-wide license to publish the Supplemental Material files. Please contact the corresponding author directly for reuse.
