# Supplementary figures and images for: Non-pharmaceutical interventions for COVID-19 transiently reduced pneumococcal and Haemophilus influenzae carriage in a cross-sectional pediatric cohort in Southampton, UK
Source: Microbiol Spectr. 2024 Jul 11;12(8):e00224-24. doi: 10.1128/spectrum.00224-24 (PMC11302307; doi:10.1128/spectrum.00224-24)

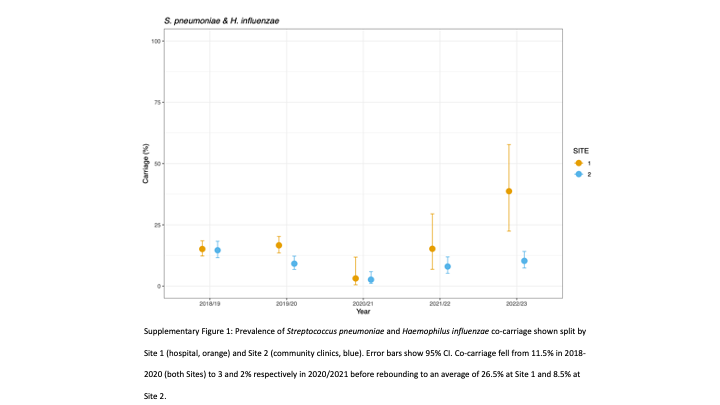

Supplement: Figure S1 — Prevalence of Streptococcus pneumoniae and Haemophilus influenzae co-carriage. [file spectrum.00224-24-s0001.tiff]

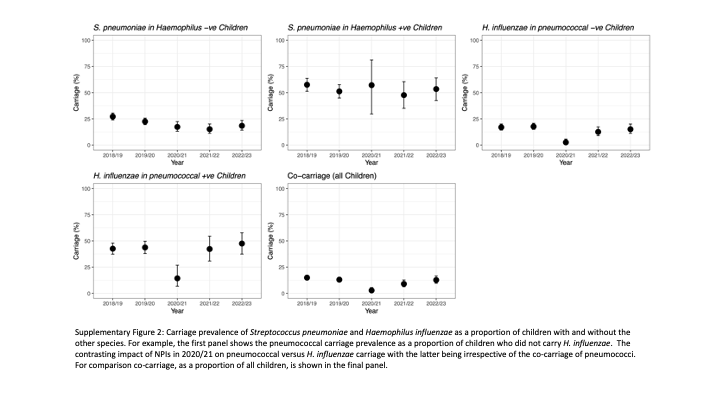

Supplement: Figure S2 — Carriage prevalence of Streptococcus pneumoniae and Haemophilus influenzae. [file spectrum.00224-24-s0002.tiff]
